# Stable integration of the Mrx1-roGFP2 biosensor to monitor dynamic changes of the mycothiol redox potential in *Corynebacterium glutamicum*

**DOI:** 10.1016/j.redox.2018.11.012

**Published:** 2018-11-17

**Authors:** Quach Ngoc Tung, Vu Van Loi, Tobias Busche, Andreas Nerlich, Maren Mieth, Johanna Milse, Jörn Kalinowski, Andreas C. Hocke, Haike Antelmann

**Affiliations:** a Freie Universität Berlin, Institute for Biology-Microbiology, D-14195 Berlin, Germany; b Center for Biotechnology (CeBiTec), Universitätsstraße 25, D-33615 Bielefeld, Germany; c Department of Internal Medicine/Infectious Diseases and Respiratory Medicine, Charité -Universitätsmedizin Berlin, D-10117 Berlin, Germany

**Keywords:** Brx, bacilliredoxin, Brx-roGFP2, bacilliredoxin-fused roGFP2 biosensor, BSH, bacillithiol, BSSB, bacillithiol disulfide, CBB, Coomassie Brilliant Blue, CLSM, confocal laser scanning microscopy, CHP, cumene hydroperoxide, DTT, dithiothreitol, ECF, extracytoplasmic function, EGT, ergothioneine, *E*_MSH_, mycothiol redox potential, Grx1-roGFP2, glutaredoxin-fused roGFP2 biosensor, GSH, glutathione, GSSG, glutathione disulfide, H_2_O_2_, hydrogen peroxide, HOCl, hypochloric acid, IPTG, isopropyl-β-D-thiogalactopyranoside, KatA, catalase, LB, Luria Bertani, LMW thiol, low molecular weight thiol, Mrx1, mycoredoxin-1, Mrx1-roGFP2, mycoredoxin-1-fused roGFP2 biosensor, MSH, mycothiol, MSSM, mycothiol disulfide, Mpx, mycothiol peroxidase, Mtr, mycothiol disulfide reductase, NaOCl, sodium hypochlorite, NEM, N-ethylmaleimide, OD_500_, optical density at 500 nm, OxD, oxidation degree, PAGE, polyacrylamide gel electrophoresis, PCR, polymerase chain reaction, RCS, reactive chlorine species, roGFP2, redox-sensitive green fluorescent protein, ROS, reactive oxygen species, SDS, sodium dodecyl sulfate, SEM, standard error of the mean, SigH, RNA polymerase sigma-H factor, TL, transmitted light, Tpx, thiol peroxidase, Trx, thioredoxin, TrxR, thioredoxin reductase, *Corynebacterium glutamicum*, Mycothiol, Mycothiol redox potential, Mrx1-roGFP2

## Abstract

Mycothiol (MSH) functions as major low molecular weight (LMW) thiol in the industrially important *Corynebacterium glutamicum*. In this study, we genomically integrated an Mrx1-roGFP2 biosensor in *C. glutamicum* to measure dynamic changes of the MSH redox potential (*E*_MSH_) during the growth and under oxidative stress. *C. glutamicum* maintains a highly reducing intrabacterial *E*_MSH_ throughout the growth curve with basal *E*_MSH_ levels of ~− 296 mV. Consistent with its H_2_O_2_ resistant phenotype, *C. glutamicum* responds only weakly to 40 mM H_2_O_2_, but is rapidly oxidized by low doses of NaOCl. We further monitored basal *E*_MSH_ changes and the H_2_O_2_ response in various mutants which are compromised in redox-signaling of ROS (OxyR, SigH) and in the antioxidant defense (MSH, Mtr, KatA, Mpx, Tpx). While the probe was constitutively oxidized in the *mshC* and *mtr* mutants, a smaller oxidative shift in basal *E*_MSH_ was observed in the *sigH* mutant. The catalase KatA was confirmed as major H_2_O_2_ detoxification enzyme required for fast biosensor re-equilibration upon return to non-stress conditions. In contrast, the peroxiredoxins Mpx and Tpx had only little impact on *E*_MSH_ and H_2_O_2_ detoxification. Further live imaging experiments using confocal laser scanning microscopy revealed the stable biosensor expression and fluorescence at the single cell level. In conclusion, the stably expressed Mrx1-roGFP2 biosensor was successfully applied to monitor dynamic *E*_MSH_ changes in *C. glutamicum* during the growth, under oxidative stress and in different mutants revealing the impact of Mtr and SigH for the basal level *E*_MSH_ and the role of OxyR and KatA for efficient H_2_O_2_ detoxification under oxidative stress.

## Highlights

• Construction of stably integrated Mrx1-roGFP2 in *C. glutamicum.*
• Highly reducing basal *E*_MSH_ of ~− 296 mV along the growth curve.
• Mtr and SigH contribute to basal *E*_MSH_ in *C. glutamicum.*
• KatA and OxyR required for *E*_MSH_ regeneration under oxidative stress.
• Live imaging revealed stable biosensor expression and fluorescence.

## Graphical abstract

**Figure:**
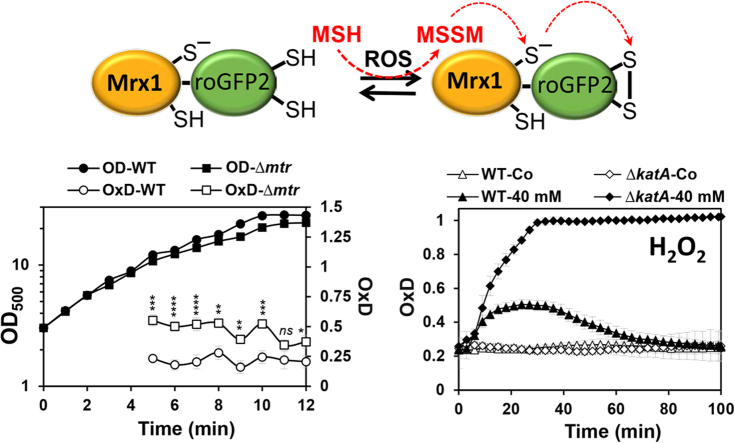

## 1 Introduction

The Gram-positive soil bacterium *Corynebacterium glutamicum* is the most important industrial platform bacterium that produces millions of tons of L-glutamate and L-lysine every year as well as other value-added products [1], [2], [3], [4]. In addition, *C. glutamicum* serves as model bacterium for the related pathogens *Corynebacterium diphtheriae and Corynebacterium jeikeium* [5]. In its natural soil habitat and during industrial production, *C. glutamicum* is exposed to reactive oxygen species (ROS), such as hydrogen peroxide (H_2_O_2_) which is generated as consequence of the aerobic lifestyle [6], [7], [8]. The low molecular weight (LMW) thiol mycothiol (MSH) functions as glutathione surrogate in detoxification of ROS and other thiol-reactive compounds in all actinomycetes, including *C. glutamicum* and mycobacteria to maintain the reduced state of the cytoplasm [9], [10], [11]. Thus, MSH-deficient mutants are sensitive to various thiol-reactive compounds, although the secreted histidine-derivative ergothioneine (EGT) also functions as alternative LMW thiol [12], [13], [14], [15], [16].

MSH is a thiol-cofactor for many redox enzymes and is oxidized to mycothiol disulfide (MSSM) under oxidative stress. The NADPH-dependent mycothiol disulfide reductase (Mtr) catalyzes the reduction of MSSM back to MSH to maintain the highly reducing MSH redox potential (*E*_MSH_) [17], [18]. Overexpression of Mtr has been shown to increase the fitness, stress tolerance and MSH/MSSM ratio during exposure to ROS, antibiotics and alkylating agents in *C. glutamicum* [19]. Under hypochloric acid (HOCl) stress, MSH functions in protein *S*-mycothiolations as discovered in *C. glutamicum, C. diphtheriae* and *Mycobacterium smegmatis* [15], [16], [20]. In *C. glutamicum,* 25 *S*-mycothiolated proteins were identified under HOCl stress that include the peroxiredoxins (Tpx, Mpx, AhpE) and methionine sulfoxide reductases (MsrA, MsrB) as antioxidant enzymes that were inhibited by *S*-mycothiolation [16], [21], [22], [23], [24], [25], [26]. The regeneration of their antioxidant activities required the mycoredoxin-1 (Mrx1)/MSH/Mtr redox pathway, but could be also coupled to the thioredoxin/ thioredoxin reductase (Trx/TrxR) pathway which both operate in de-mycothiolation [9], [10], [27]. Detailed biochemical studies on the redox-regulation of antioxidant and metabolic enzymes (Tpx, Mpx, MsrA, GapDH) showed that both, the Mrx1 and Trx pathways function in de-mycothiolation at different kinetics. Mrx1 was much faster in regeneration of GapDH and Mpx activities during recovery from oxidative stress compared to the Trx pathway [20], [21], [23], [24], [25], [26].

The enzymes for MSH biosynthesis and the Trx/TrxR systems are under control of the alternative extracytoplasmic function (ECF) sigma factor SigH which is sequestered by its cognate redox-sensitive anti sigma factor RshA in non-stressed cells [28], [29], [30]. RshA is oxidized under disulfide stress leading to structural changes and relief of SigH to initiate transcription of the large SigH disulfide stress regulon [16], [31], [32], [33]. In addition, the LysR-type transcriptional repressor OxyR plays a major role in the peroxide response in *C. glutamicum* which controls genes encoding antioxidant enzymes for H_2_O_2_ detoxification and iron homeostasis, such as the catalase (*katA*), two miniferritins (*dps, ftnA*), the Suf machinery and ferrochelatase (*hemH*) [30], [34]. Thus, SigH and OxyR can be regarded as main regulatory systems for the defense under disulfide and oxidative stress to maintain the redox balance in actinomycetes.

The standard thiol-redox potential of MSH was previously determined with biophysical methods as *E*^0′^(MSSM/MSH) of − 230 mV which is close to that of glutathione (GSH) [35]. However, Mrx1 was also recently fused to redox-sensitive green fluorescent protein (roGFP2) to construct a genetically encoded Mrx1-roGFP2 redox biosensor for dynamic measurement of *E*_MSH_ changes inside mycobacterial cells. *E*_MSH_ values of ~-300 mV were calculated using the Mrx1-roGFP2 biosensor in mycobacteria that were much lower compared to values obtained with biophysical methods [35], [36]. This Mrx1-roGFP2 biosensor was successfully applied for dynamic *E*_MSH_ measurements in the pathogen *Mycobacterium tuberculosis* (*Mtb*). Using Mrx1-roGFP2, *E*_MSH_ changes were studied in drug-resistant *Mtb* isolates, during intracellular replication and persistence in the acidic phagosomes of macrophages [36], [37], [38]. Mrx1-roGFP2 was also applied as tool in drug research to screen for ROS-generating anti-tuberculosis drugs or to reveal the mode of action of combination therapies based on *E*_MSH_ changes [36], [39], [40], [41]. The *Mtb* population exhibited redox heterogeneity of *E*_MSH_ during infection inside macrophages which was dependent on sub-vacuolar compartments and the cytoplasmic acidification controlled by WhiB3 [36], [38]. Thus, application of the Mrx1-roGFP2 biosensor provided novel insights into redox changes of *Mtb*. However, Mrx1-roGFP2 has not been applied in the industrial platform bacterium *C. glutamicum*.

In this work, we designed a genetically encoded Mrx1-roGFP2 biosensor that was genomically integrated and expressed in *C. glutamicum*. The biosensor was successfully applied to measure dynamic *E*_MSH_ changes during the growth, under oxidative stress and in various mutant backgrounds to study the impact of antioxidant systems (MSH, KatA, Mpx, Tpx) and their major regulators (OxyR, SigH) under basal and oxidative stress conditions. Our results revealed a highly reducing basal *E*_MSH_ of ~-296 mV that is maintained throughout the growth of *C. glutamicum*. H_2_O_2_ stress had only little effect on *E*_MSH_ changes in the wild type due to its H_2_O_2_ resistance, which was dependent on the catalase KatA supporting its major role for H_2_O_2_ detoxification. Confocal imaging further confirmed equal Mrx1-roGFP2 fluorescence in all cells indicating that the biosensor strain is well suited for industrial application to quantify *E*_MSH_ changes in *C. glutamicum* at the single cell level.

## 2 Materials and methods

### 2.1 Bacterial strains and growth conditions

Bacterial strains, plasmids and primers are listed in Tables S1 and S2. For cloning and genetic manipulation, *Escherichia coli* was cultivated in Luria Bertani (LB) medium at 37 °C. The *C. glutamicum* ATCC13032 wild type as well as the Δ*mshC,* Δ*mtr,* Δ*oxyR,* Δ*sigH*, Δ*katA,* Δ*mpx,* Δ*tpx* and Δ*mpx tpx* mutant strains were used in this study for expression of the Mrx1-roGFP2 biosensor which are described in Table S1. All *C. glutamicum* strains were cultivated in heart infusion medium (HI; Difco) at 30 °C overnight under vigorous agitation. The overnight culture was inoculated in CGC minimal medium supplemented with 1% glucose to an optical density at 500 nm (OD_500_) of 3.0 and grown until OD_500_ of 8.0 for stress exposure as described [16]. *C. glutamicum* mutants were cultivated in the presence of the antibiotics nalidixic acid (50 μg/ml) and kanamycin (25 μg/ml).

### 2.2 Construction, expression and purification of His-tagged Mrx1-roGFP2 protein in *E. coli*

The *mrx1* gene (*cg0964*) was amplified from chromosomal DNA of *C. glutamicum* ATCC13032 by PCR using the primer pair Cgmrx1-roGFP2-*Nde*I-FOR and pQE60-Cgmrx1-roGFP2-*Spe*I-REV. The PCR product was digested with *Nde*I and *Spe*I and cloned into plasmid pET11b-*brx-roGFP2* [42] to exchange the *brx* sequence by *mrx1* with generation of plasmid pET11b-*mrx1-roGFP2* **(**Table S1**)**. The correct sequence was confirmed by PCR and DNA sequencing.

The *E. coli* BL21 (DE3) *plysS* expression strain containing the plasmid pET11b-*mrx1-roGFP2* was grown in 1 l LB medium until OD_600_ of 0.6 at 37 °C, followed by induction with 1 mM IPTG (isopropyl-β-D-thiogalactopyranoside) for 16 h at 25 °C. Recombinant His_6_-tagged Mrx1-roGFP2 protein was purified using His Trap™ HP Ni-NTA columns (5 ml; GE Healthcare, Chalfont St Giles, UK) and the ÄKTA purifier liquid chromatography system (GE Healthcare) according to the instructions of the manufacturer (USB). The purified protein was dialyzed against 10 mM Tris-HCl (pH 8.0), 100 mM NaCl and 30% glycerol and stored at − 80 °C. Purity of the protein was analyzed after sodium dodecyl sulfate-polyacrylamide gel electrophoresis (SDS-PAGE) and Coomassie brilliant blue (CBB) staining.

### 2.3 Construction of *katA, mtr, mpx* and *tpx* deletion mutants in *C. glutamicum*

The vector pK18*mobsacB* was used to create marker-free deletions in *C. glutamicum* (1). The gene-SOEing method of Horton (2) was used to construct pK18*mobsacB* derivatives to perform allelic exchange of the *katA* and *mtr* genes in the chromosome of *C. glutamicum* ATCC13032 using the primers listed in Table S2. The constructs include the *katA* and *mtr* genes with flanking regions and internal deletions (Δ*katA* [1555 bp] and Δ*mtr* [1382 bp]). The pK18*mobsacB* derivatives were sub-cloned in *E. coli* JM109 (Table S1) and transformed into *C. glutamicum* ATCC13032. The pK18*mobsacB*::Δ*tpx* plasmid containing the *tpx* flanking regions was constructed previously (3) and transformed into the *C. glutamicum* Δ*mpx* mutant (3). The gene replacement in the chromosome of *C. glutamicum* ATCC13032 resulted in Δ*katA* and Δ*mtr* single deletion mutants and the gene replacement of *tpx* in the chromosome of *C. glutamicum* Δ*mpx* resulted in the *C. glutamicum* Δ*mpx tpx* double deletion mutant. The deletions were confirmed by PCR using the primers in Table S2.

### 2.4 Construction of *C. glutamicum* Mrx1-roGFP2 biosensor strains

For construction of the genomically integrated Mrx1-roGFP2 biosensor, a 237 bp fragment of *mrx1* (*cg0964*) was fused to *roGFP2* containing a 30-amino acid linker (GGSGG)_6_ under control of the strong P_*tuf*_ promoter of the *C. glutamicum tuf* gene encoding the translation elongation factor EF-Tu. The P_*tuf*_-Mrx1-roGFP2 fusion was codon-optimized, synthesized with flanking *Mun*I and *Xho*I restriction sites and sub-cloned into PUC-SP by Bio Basic resulting in PUC-SP::P_*tuf*_*-mrx1-roGFP2*. For genomic integration of the biosensor into the *cg1121*-*cg1122* intergenic region of *C. glutamicum* (Table S1), the vector pK18*mobsacB*-*cg1121-cg1122* was used [43], kindly provided by Julia Frunzke, Forschungszentrum Jülich. The vector was PCR amplified with primers pk18_MunI and pk18_XhoI to swap the restrictions sites. After digestion of the pk18*mobsacB*-*cg1121-cg1122* PCR product and the PUC-SP::*P*_*tuf*_*-mrx1-roGFP2* plasmid with *Mun*I and *Xho*I, both digestion products were ligated to obtain pK18*mobsacB-cg1121-cg1121*-P_*tuf*_*-mrx1-roGFP2.* The resulting plasmid was sequenced with biosensor_seq_primer_1 and biosensor_seq_primer_2. Transfer of the plasmid into *C. glutamicum* strains (Table S1) was performed by electroporation and screening for double homologous recombination events using the conditional lethal effect of the *sacB* gene as described [16], [43]. Correct integration of P_*tuf*_*-mrx1-roGFP2* into the *cg1121*-*cg1122* intergenic region was verified by colony PCR using 2 primer pairs (pk18_INT_Cg_Test_rev, pk18_INT_Cg_Test_fwd and FUB_7_seq_wo_linker_fwd; FUB_8_seq_wo_linker_rev) (Table S2).

The Mrx1-roGFP2 biosensor was further cloned into the *E. coli-C. glutamicum* shuttle vector pEKEx2 for ectopic expression of Mrx1-roGFP2 under the IPTG-inducible *tac* promoter. The *mrx1-roGFP2* fusion was amplified from plasmid pET11b-*mrx1-roGFP2* using primer pair pEKEx2-Cgmrx1-*Bam*HI-For and pEKEx2-roGFP2-*Kpn*I-Rev (Table S2). The PCR product and plasmid pEKEx2 were digested with *BamH*I and *Kpn*I, followed by ligation to generate plasmid pEKEx2-*mrx1-roGFP2*. The resulting plasmid was cloned in *E. coli*, sequenced and electroporated into *C. glutamicum*. Induction of the *C. glutamicum* strain expressing pEKEx2-encoded Mrx1-roGFP2 was performed with 1 mM IPTG.

### 2.5 Characterization of recombinant Mrx1-roGFP2 biosensor *in vitro*

The purified Mrx1-roGFP2 protein was reduced with 10 mM dithiothreitol (DTT) for 20 min, desalted with Micro-Bio spin columns (Bio-Rad), and diluted to a final concentration of 1 µM in 100 mM potassium phosphate buffer, pH 7.0. The oxidation degree (OxD) of the biosensor was determined by calibration to fully reduced and oxidized probes which were generated by treatment of the probes with 10 mM DTT and 5 mM diamide for 5 min, respectively [42]. The thiol disulfides and oxidants were injected into the microplate wells (BD Falcon 353219) 60 s after the start of measurements. Emission was measured at 510 nm after excitation at 400 and 488 nm using the CLARIOstar microplate reader (BMG Labtech) with the Control software version 5.20 R5. Gain setting was adjusted for each excitation maximum. The data were analyzed using the MARS software version 3.10 and exported to Excel. Each *in vitro* measurement was performed in triplicate.

### 2.6 Measurements of Mrx1-roGFP2 biosensor oxidation in *C. glutamicum in vivo*

*C. glutamicum* wild type and mutant strains expressing stably integrated Mrx1-roGFP2 were grown overnight in HI medium and inoculated into CGC medium with 1% glucose to a starting OD_500_ of 3.0. For stress experiments, the strains were cultivated for 8 h until they have reached an OD_500_ of 14–16. Cells were harvested by centrifugation, washed twice with CGC minimal medium, adjusted to an OD_500_ of 40 in CGC medium and transferred to the microplate reader. Aliquots were treated for 15 min with 10 mM DTT and 20 mM cumene hydroperoxide (CHP) for fully reduced and oxidized controls, respectively. Injection of the oxidants was performed 5 min after the start of microplate reader measurements.

For the OxD measurements along the growth curves, cells were harvested by centrifugation at different time points and washed in 100 mM potassium phosphate buffer, pH 7.0. Aliquots were treated with 20 mM CHP and 10 mM DTT for fully reduced and oxidized controls, respectively. Samples and controls were incubated with 10 mM N-ethylmaleimide (NEM) to block free thiols and transferred to microplate wells. The Mrx1-roGFP2 biosensor fluorescence emission was measured at 510 nm after excitation at 400 and 488 nm using the CLARIOstar microplate reader (BMG Labtech). The OxD of biosensor was calculated for each sample and normalized to fully reduced and oxidized controls as described previously [42], [44] based to the following Eq. (1).

(1) $$\mathrm{OxD}=\frac{{I400}_{\mathrm{sample}}{\times \;{I488}_{\mathrm{red}}-\;{I400}_{\mathrm{red}}\times \;{I488}_{\mathrm{sample}}}_{\;}}{{{I400}_{\mathrm{sample}}\times \;{I488}_{\mathrm{red}}-\;{I400}_{\mathrm{sample}}\times \;{I488}_{\mathrm{ox}}\;+\;{I400}_{\mathrm{ox}}\times {I488}_{\mathrm{sample}}-\;{I400}_{\mathrm{red}}\times \;{I488}_{\mathrm{sample}}}^{\;}}$$

The values of *I*400_sample_ and *I*488_sample_ are the observed fluorescence excitation intensities at 400 and 488 nm, respectively. The values of *I*400_red_, *I*488_red_, *I*400_ox_ and *I*488_ox_ represent the fluorescence intensities of fully reduced and oxidized controls, respectively.

Based on the OxD and $E_{\mathrm{roGFP}2}^{o\prime }$ = − 280 mV [45], the MSH redox potential was calculated according to the Nernst Eq. (2) as follows:

(2) $$E_{\mathrm{MSH}}=E_{\mathrm{roGFP}2}=E_{\mathrm{roGFP}2}^{o'}-\left(\frac{\mathrm{RT}}{2F}\right)*\mathrm{In}\left(\frac{1-\mathrm{OxD}}{\mathrm{OxD}}\right)$$

### 2.7 Confocal laser scanning microscopy of *Mrx1-roGFP2 biosensor strains*

*C. glutamicum* wild type expressing Mrx1-roGFP2 was grown in HI medium for 48 h, exposed to 80 mM H_2_O_2_ for different times and washed in potassium phosphate buffer, pH 7.0. Cells were blocked with 10 mM NEM, and imaged using a LSM 780 confocal laser-scanning microscope with a 63 × /1.4 NA Plan-Apochromat oil objective controlled by the Zen 2012 software (Carl-Zeiss, Jena, Germany). Fluorescence excitation was performed at 405 and 488 nm with laser power adjustment to 15% and 25%, respectively. For both excitation wavelengths, emission was collected between 491 and 580 nm. Fully reduced and oxidized controls were prepared with 10 mM DTT and 10 mM diamide, respectively. Images were analyzed by the Zen 2 software and Fiji/ImageJ [42], [46]. Fluorescent intensities were measured after excitation at 405 and 488 nm and the images false-colored in red and green, respectively. Auto-fluorescence was recorded and subtracted. Quantification of the OxD and *E*_MSH_ values was performed based on the 405/488 nm excitation ratio of mean fluorescence intensities as described [42], [46].

## 3 Results

### 3.1 The Mrx1-roGFP2 biosensor of *C. glutamicum* responds most specifically to MSSM *in vitro*

**Fig. 1:**
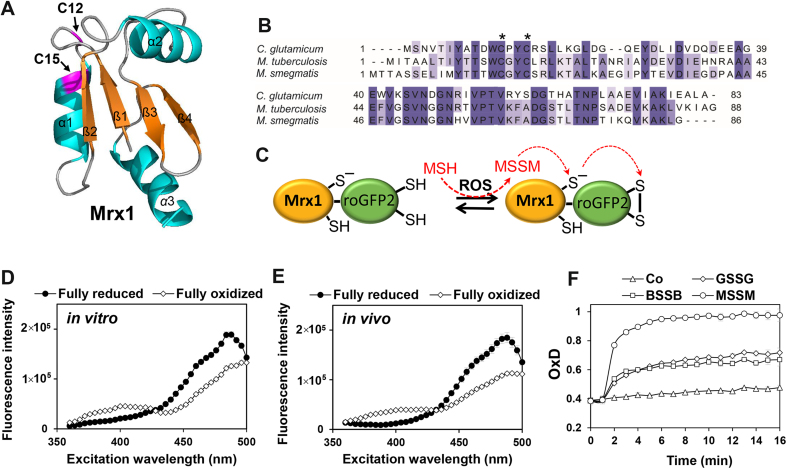
**Structure and alignment of Mrx1 homologs, principle and specific response of the Mrx1-roGFP2 biosensor to MSSM. (A)** The Mrx1 structure of *C. glutamicum* was modelled using SWISS-MODEL (https://swissmodel.expasy.org/) and visualized with PyMol using the template of *M. tuberculosis* Rv3198A (PDB code: 2LQO). The Cys12 active site and Cys15 resolving site of the CXXC motif of Mrx1 are labelled with arrows. **(B)** The Mrx1 homologs Cg0964 of *C. glutamicum*, Rv3198A of *M. tuberculosis* and MSMEG_1947 of *M. smegmatis* were aligned with ClustalW2 and presented in Jalview. Intensity of the blue color gradient is based on 50% identity. Conserved Cys residues are marked with asterisks. **(C)** The principle of the Mrx1-roGFP2 biosensor oxidation is shown. Under ROS stress, MSH is oxidized to MSSM which reacts with Mrx1 to *S*-mycothiolated Mrx1. MSH is transferred from Mrx1 to the roGFP2 moiety leading to *S*-mycothiolated roGFP2 which is rearranged to the roGFP2 disulfide. The roGFP2 disulfide leads to a structural change resulting in ratiometric changes of the 400 and 488 excitation maxima of Mrx1-roGFP2. **(D, E)** The ratiometric response of the Mrx1-roGFP2 biosensor in the reduced and oxidized state *in vitro***(D)** and after expression in *C. glutamicum in vivo***(E)**. For fully reduced and oxidized Mrx1-roGFP2, 10 mM DTT and 5 mM diamide were used *in vitro* as well as 10 mM DTT and 20 mM CHP *in vivo* (n = 5). The fluorescence excitation spectra were monitored using the microplate reader. **(F)** The purified Mrx1-roGFP2 biosensor (1 µM) responds most strongly to 100 µM of MSSM, but only weakly to BSSB and GSSG *in vitro* (n = 3). The thiol disulfides were injected into the microplate wells 60 s after the start of the measurements of the Mrx1-roGFP2 biosensor response. The control (Co) indicates the measurement of the Mrx1-roGFP2 biosensor response without thiol-disulfides. The OxD was calculated based on the 400/488 nm excitation ratio with emission measured at 510 nm. Mean values and standard error of the mean (SEM) are shown in all graphs.

Previous studies have revealed a specific response of the Mrx1-roGFP2 biosensor to MSSM *in vitro*, which was based on a fusion of mycobacterial Mrx1 to roGFP2 [36]. Here we aimed to engineer a related Mrx1-roGFP2 biosensor for the MSH-producing industrially important bacterium *C. glutamicum*. Mrx1 (Cg0964) of *C. glutamicum* exhibits a similar redox-active CxxC motif and shares 46.8% and 42.1% sequence identity with Mrx1 homologs of *M. tuberculosis* H37Rv (Rv3198A) and *M. smegmatis* mc^2^155 (MSMEG_1947), respectively (Fig. 1**AB**) [27]. The principle of the Mrx1-roGFP2 biosensor to measure intrabacterial *E*_MSH_ changes was shown previously [14], [36]. MSSM reacts with Mrx1 to form *S*-mycothiolated Mrx1, followed by the transfer of the MSH moiety to roGFP2 which rearranges to the roGFP2 disulfide resulting in ratiometric changes of the 400/488 excitation ratio [14], [36] (Fig. 1C).

Mrx1 of *C. glutamicum* was fused to roGFP2 and first purified as His-tagged Mrx1-roGFP2 protein to verify the specific Mrx1-roGFP2 biosensor response to MSSM *in vitro*. In addition, Mrx1-roGFP2 was integrated into the genome of *C. glutamicum* wild type in the intergenic region between *cg1121-cg1122* and placed under control of the strong P_*tuf*_ promoter using the pK18*mobsacB-int* plasmid as constructed previously [43]. First, the Mrx1-roGFP2 biosensor response of the purified biosensor and of the stably integrated Mrx1-roGFP2 fusion were compared under fully reduced (DTT) and fully oxidized (diamide) conditions. The Mrx1-roGFP2 biosensor fluorescence excitation spectra were similar under *in vitro* and *in vivo* conditions exhibiting the same excitation maxima at 400 and 488 nm for fully reduced and oxidized probes (Fig. 1**DE**). Thus, the Mrx1-roGFP2 probe is well suited to monitor dynamic *E*_MSH_ changes during the growth and under oxidative stress in *C. glutamicum*. In addition, it was verified that purified Mrx1-roGFP2 reacts very fast and most strongly to low levels of 100 µM MSSM, although weaker responses were also observed with bacillithiol disulfide (BSSB) and glutathione disulfide (GSSG) which are, however, not physiologically relevant for *C. glutamium* (Fig. 1**F**).

**Fig. 2:**
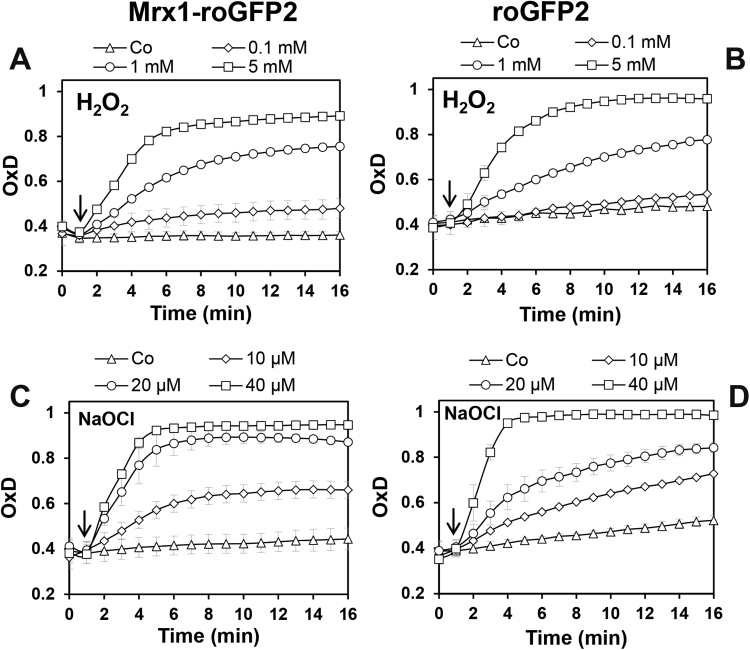
**The response of the purified Mrx1-roGFP2 and roGFP2 biosensors to H**_**2**_**O**_**2**_**and NaOCl*****in vitro***. Purified Mrx1-roGFP2 and roGFP2 probes (1 µM) were treated with increasing concentrations of 0.1–5 mM H_2_O_2_**(A, B)** and 10–40 µM NaOCl **(C, D)**, respectively. The oxidants were injected into the microplate wells 60 s after the start of the measurements of the Mrx1-roGFP2 biosensor response as indicated by arrows. The control (Co) indicates the measurement of the Mrx1-roGFP2 and roGFP2 response without oxidants. The OxD was calculated based on the 400/488 nm excitation ratios with emission at 510 nm and related to the fully oxidized (5 mM diamide) and reduced controls (10 mM DTT). Mean values of 5 independent experiments are shown and error bars represent the SEM.

Furthermore, we assessed the direct response of Mrx1-roGFP2 and unfused roGFP2 to the oxidants H_2_O_2_ and NaOCl to compare the sensitivities of the probes for direct oxidation (Fig. 2). This was important since a previous study showed a high sensitivity of fused Grx-roGFP2 and roGFP2-Orp1 to 10-fold molar excess of 2 µM NaOCl [47]. In our *in vitro* experiments, the Mrx1-roGFP2 and roGFP2 probes did not respond to 100 µM H_2_O_2_ as in previous studies. Only 1–5 mM H_2_O_2_ lead to a direct oxidation of both probes with a faster response of the Mrx1-roGFP2 fusion. Both probes were rapidly oxidized by 10–40 µM NaOCl *in vitro*, and again Mrx1-roGFP2 was more sensitive to thiol-oxidation by NaOCl compared to unfused roGFP2 (Fig. 2). The rapid oxidation of roGFP2 and fused roGFP2 biosensors to low levels of HOCl is in agreement with previous studies [47] and was also observed using the Brx-roGFP2 biosensor in *S. aureus* [42]. The higher sensitivity of fused roGFP2 biosensors (Brx-roGFP2, Mrx1-roGFP2) to NaOCl indicates that the redox active Cys residues of Brx or Mrx1 are more susceptible for thiol-oxidation compared to the thiols of roGFP2. In conclusion, our Mrx1-roGFP2 probe is highly specific to low levels of MSSM. The response of Mrx1-roGFP2 to higher levels of 1 mM H_2_O_2_ *in vitro* are not expected to occur inside *C. glutamicum* cells due to its known H_2_O_2_ resistance mediated by the highly efficient catalase.

### 3.2 The intracellular redox balance was affected in mutants with defects of MSH, Mtr and SigH

**Fig. 3:**
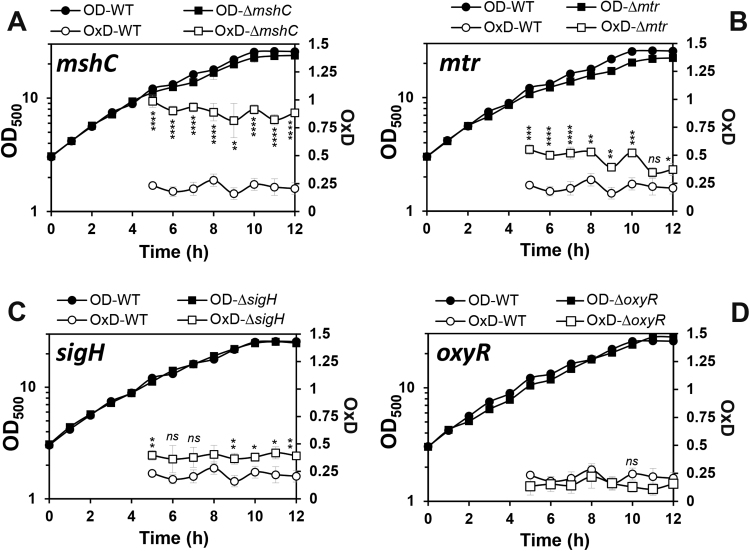
**Deletions of*****mshC, mtr*****and*****sigH*****affected the basal*****E***_**MSH**_**during the growth of*****C. glutamicum***. The basal level of *E*_MSH_ was measured using Mrx1-roGFP2 along the growth curve in *C. glutamicum* wild type and in ∆*mshC***(A)**, ∆*mtr***(B)**, ∆*sigH***(C)** and ∆*oxyR***(D)** mutants. The basal *E*_MSH_ showed an oxidative shift in the ∆*mshC,* ∆*mtr and* ∆*sigH* mutants, but not in the ∆*oxyR* mutant **(D)**. OxD was calculated based on the 400/488 nm excitation ratios with emission at 510 nm and related to the fully oxidized and reduced controls. Mean values and SEM of four independent experiments are shown and *p*-values were calculated by the Student's unpaired two-tailed *t*-test by the graph prism software (^ns^p > 0.05; *p<0.05; **p<0.01; ***p<0.001; and ****p<0.0001).

**Fig. 4:**
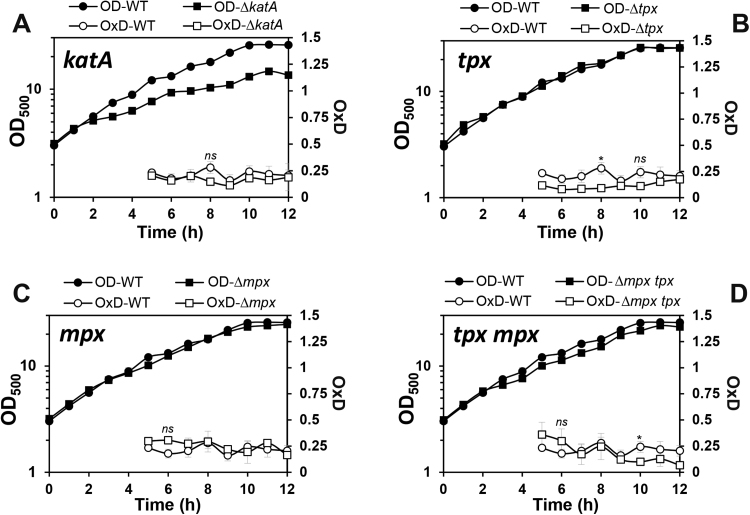
**The absence of the antioxidant enzymes KatA, Tpx and Mpx has no influence on the basal level*****E***_**MSH**_**during the growth of*****C. glutamicum*****.** The basal level of *E*_MSH_ was measured using the Mrx1-roGFP2 along the growth curve in *C. glutamicum* wild type and ∆*katA***(A)**, ∆*tpx***(B)**, ∆*mpx***(C)** and ∆*tpx mpx***(D)** mutants, but was not affected compared to the wild type. OxD was calculated based on the 400/488 nm excitation ratios with emission at 510 nm and related to the fully oxidized and reduced controls. Mean values and SEM of four independent experiments are shown and *p*-values were calculated by the Student's unpaired two-tailed *t*-test by the graph prism software (^ns^p > 0.05; *p < 0.05; **p < 0.01; ***p < 0.001; and ****p < 0.0001).

Next, we applied the genomically expressed Mrx1-roGFP2 biosensor to monitor the perturbations of basal level *E*_MSH_ along the growth curve in various *C. glutamicum* mutant backgrounds, which had deletions of major antioxidant systems (MSH, Mtr, KatA, Tpx, Mpx) and redox-sensing regulators (OxyR, SigH) (Fig. 3, Fig. 4). The oxidation degree was calculated in *C. glutamicum* wild type and mutants during the 5–12 h time points representing the log phase and transition to stationary phase in defined CGC medium. The biosensor oxidation of each *C. glutamicum* sample was normalized between 0 and 1 based on the fully reduced (DTT) and oxidized (CHP) controls. It is interesting to note, that *C. glutamicum* wild type cells maintained a highly reducing and stable *E*_MSH_ of ~-296 mV with little fluctuations during the log and stationary phase (Table S3). Thus, this basal level *E*_MSH_ of *C. glutamicum* is very similar to that measured in *M*. smegmatis previously (*E*_MSH_ of ~−300) [36].

In agreement with previous studies of bacillithiol (BSH)- and GSH-deficient mutants, the absence of MSH resulted in constitutive oxidation of the Mrx1-roGFP2 biosensor in the *mshC* mutant (Fig. 3**A**). This indicates an impaired redox state in the *mshC* mutant and the importance of MSH as major LMW thiol to maintain the redox balance in *C. glutamicum* (Fig. 3**A**). We hypothesize that increased levels of ROS may lead to constitutive biosensor oxidation in the MSH-deficient mutant since the *mshC* mutant had a H_2_O_2_-sensitive phenotype in previous studies [48]. The high MSH/MSSM redox balance is maintained by the NADPH-dependent mycothiol disulfide reductase Mtr which reduces MSSM back to MSH [9]. The importance of Mtr to maintain a reduced *E*_MSH_ was also supported by our biosensor measurements which revealed an oxidative shift in *E*_MSH_ to −280.2 mV in the *mtr* mutant during all growth phases (Fig. 3**B,** Table S3).

The alternative ECF sigma factor SigH controls a large disulfide stress regulon mainly involved in the redox homeostasis, including genes for thioredoxins and thioredoxin reductases (TrxAB), mycoredoxin-1 (Mrx1) and genes for MSH biosynthesis and recycling (MshA, Mca, Mtr) [9], [28], [29], [32]. The *C. glutamicum sigH* mutant showed an increased sensitivity to ROS and NaOCl stress [16], [28], [29]. Mrx1-roGFP2 biosensor measurements confirmed a slightly more oxidized *E*_MSH_ of − 286 mV in the *sigH* mutant supporting the regulatory role of SigH for the redox balance (Fig. 3**C,** Table S3). However, the oxidative *E*_MSH_ shift was lower in the *sigH* mutant compared to the *mtr* mutant. In conclusion, our Mrx1-roGFP2 biosensor results document the important role of MSH, Mtr and SigH to maintain the redox homeostasis in *C. glutamicum* during the growth.

In addition to MSH, *C. glutamicum* encodes many antioxidant enzymes that are involved in H_2_O_2_ detoxification and confer strong resistance of *C. glutamicum* to millimolar levels of H_2_O_2_. The H_2_O_2_ scavenging systems in *C. glutamicum* are the major vegetative catalase (KatA) and the peroxiredoxins (Tpx, Mpx). The catalase is highly efficient for detoxification at high H_2_O_2_ levels while Tpx and Mpx are more involved in reduction of physiological low levels of H_2_O_2_ generated during the aerobic growth [49]. In *C. glutamicum*, expression of *katA* is induced by H_2_O_2_ and controlled by the redox-sensing OxyR repressor which is inactivated under H_2_O_2_ stress [34]. Thus, the *oxyR* mutant exhibits increased H_2_O_2_ resistance due to constitutive derepression of *katA* [34]. Here, we were interested in the contribution of OxyR, and the antioxidant enzymes KatA, Tpx and Mpx to maintain the reduced basal level *E*_MSH_ in *C. glutamicum*. In all mutants with deletions of *oxyR*, *katA*, *tpx* and *mpx*, the basal level of *E*_MSH_ was still highly reducing and comparable to the wild type during different growth phases (Fig. 3**D,** Fig. 4**A–D,** Table S3). Thus, we can conclude that the major antioxidant enzymes for H_2_O_2_ detoxification (KatA, Mpx and Tpx) do not contribute to the reduced basal *E*_MSH_ level in *C. glutamicum* during aerobic growth. These results further point to the main roles of these H_2_O_2_ scavenging systems under conditions of oxidative stress to recover the reduced state of *E*_MSH_ which was investigated in the next section.

### 3.3 Mrx1-roGFP2 biosensor responses in *C. glutamicum* under oxidative stress *in vivo*

**Fig. 5:**
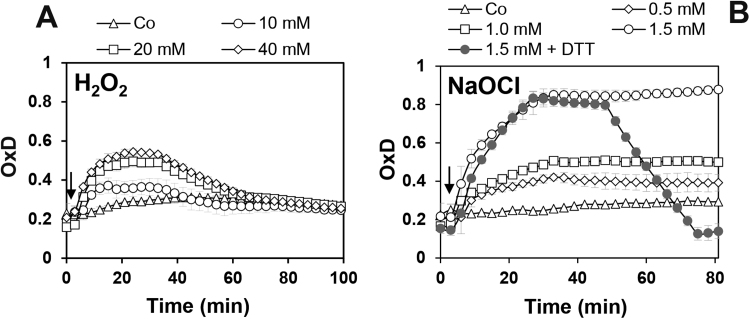
**The Mrx1-roGFP2 biosensor responds weakly to H**_**2**_**O**_**2**_**and strongly to NaOCl in*****C. glutamicum*****wild type cells**. The Mrx1-roGFP2 biosensor was weakly oxidized by 10–40 mM H_2_O_2_ in *C. glutamicum* wild type (*p* = 0.0002 at 20 mM H_2_O_2_; *p* < 0.0001 at 40 mM H_2_O_2_) **(A)**, but rapidly and fully by low doses of 0.5–1.5 mM NaOCl (*p* = 0.007 at 0.5 mM NaOCl; *p* = 0.0004 at 1.0 mM NaOCl; *p* < 0.0001 at 1.5 mM NaOCl) **(B).** While cells could recover the reduced state after 50 min of H_2_O_2_ exposure **(A)**, regeneration of Mrx1-roGFP2 was not possible in NaOCl-stressed cells **(B)**. To analyze the reversibility of Mrx1-roGFP2 oxidation in NaOCl-treated cells, 10 mM DTT was added 45 min after NaOCl exposure resulting in recovery of reduced *E*_MSH_**(B).** Mean values and SEM of three independent experiments are shown in all graphs and *p*-values are calculated by a Student's unpaired two-tailed *t*-test by the graph prism software. The addition of oxidants to *C. glutamicum* cells was performed 5 min after the start of the measurements and is indicated by arrows. The control (Co) denotes the response of the Mrx1-roGFP2 probe inside *C. glutamicum* wild type cells in the absence of oxidants.

Next, we were interested to determine the kinetics of Mrx1-roGFP2 biosensor oxidation in *C. glutamicum* under H_2_O_2_ and NaOCl stress and the recovery of reduced *E*_MSH_. *C. glutamicum* can survive even 100 mM H_2_O_2_ without killing effect which depends on the very efficient catalase KatA [34]. In accordance with the H_2_O_2_ resistant phenotype, the Mrx1-roGFP2 biosensor did not respond to 10 mM H_2_O_2_ in *C. glutamicum* wild type cells and was only weakly oxidized by 40 mM H_2_O_2_ (Fig. 5**A**). *C. glutamicum* cells were able to recover the reduced *E*_MSH_ within 40–60 min after H_2_O_2_ treatment. Importantly, even 100 mM H_2_O_2_ did not further enhance the biosensor oxidation degree, indicating highly efficient antioxidant systems (data not shown).

In contrast, *C. glutamicum* was more sensitive to sub-lethal doses of NaOCl stress and showed a moderate biosensor oxidation by 0.5–1 mM NaOCl, while 1.5 mM NaOCl resulted in the fully oxidation of the probe. Moreover, cells were unable to regenerate the reduced basal level of *E*_MSH_ within 80 min after NaOCl exposure, which could be only restored with 10 mM DTT **(**Fig. 5**B)**.

**Fig. 6:**
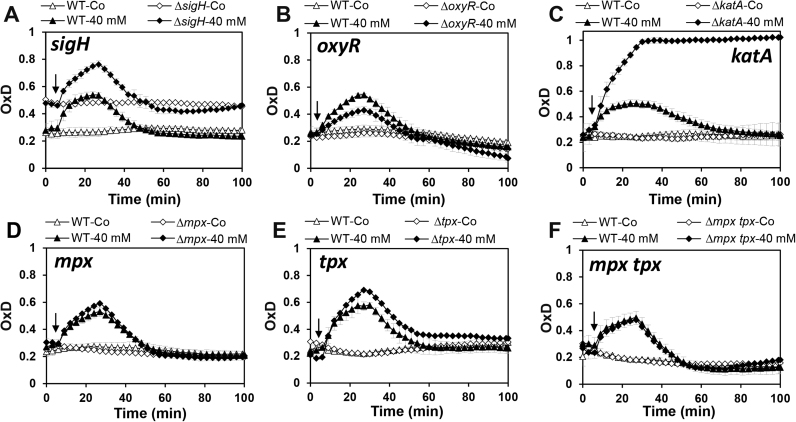
**Kinetics of H**_**2**_**O**_**2**_**detoxification in*****C. glutamicum*****mutants deficient for redox-regulators (OxyR, SigH) or antioxidant enzymes (KatA, Mpx, Tpx).** The Mrx1-roGFP2 biosensor response and kinetics of recovery was analyzed under 40 mM H_2_O_2_ stress in *C. glutamicum* wild type and mutants deficient for the disulfide stress regulatory sigma factor SigH **(A)**, the peroxide-sensitive repressor OxyR **(B)** and the catalases and peroxiredoxins for H_2_O_2_ detoxification (KatA, Mpx, Tpx) **(C-F)**. The *sigH* mutant showed a higher *E*_MSH_ basal level of *E*_MSH_, but the response and recovery under H_2_O_2_ stress was similar to the wild type **(A)**. The constitutive derepression of *katA* in the *oxyR* mutant resulted in a lower Mrx1-roGFP2 biosensor response under H_2_O_2_ stress (*p* = 0.006 WT versus *oxyR* H_2_O_2_) **(B)**. The catalase KatA is essential for H_2_O_2_ detoxification as revealed by the strong oxidation increase of the *katA* mutant and the lack of regeneration of reduced *E*_MSH_ (*p* *<* 0.0001 WT versus *katA* H_2_O_2_) **(C)**. The Mrx1-roGFP2 biosensor response of the *tpx* mutant was only slightly increased under H_2_O_2_ stress (*p* = 0.0017 WT versus *tpx* H_2_O_2_) **(E)**, but not in *mpx* and *mpx tpx* mutants (*p* = 0.7981 or *p* = 0.9489 WT versus *tpx* or *mpx tpx* H_2_O_2_) **(D, F)**. Mean values and SEM of three independent experiments are shown in all graphs and *p*-values are obtained by a Student's unpaired two-tailed *t*-test by the graph prism software. The addition of oxidants to *C. glutamicum* wild type and mutant cells was performed 5 min after the start of the measurements and is indicated by arrows. The control (Co) shows the response of the Mrx1-roGFP2 probe inside *C. glutamicum* wild type and mutant cells without H_2_O_2_ treatment.

Since H_2_O_2_ is the more physiological oxidant in *C. glutamicum*, we studied the biosensor response under 40 mM H_2_O_2_ stress in the various mutants deficient for MSH and Mtr, antioxidant enzymes (KatA, Mpx, Tpx) and redox regulators (SigH, OxyR). The *sigH* mutant showed an increased basal level of *E*_MSH_ of ~-286 mV as noted earlier (Fig. 3**C**), but a similar oxidation increase with 40 mM H_2_O_2_ and recovery of the reduced state after 40 min compared to the wild type (Fig. 6**A**). The similar kinetics of biosensor oxidation and regeneration in wild type and *sigH* mutant cells may indicate that MSH is not directly involved in H_2_O_2_ detoxification. In contrast, the *oxyR* mutant showed a lower H_2_O_2_ response than the wild type, but required the same time of 40 min for recovery of the reduced state of *E*_MSH_ (Fig. 6**B**). The derepression of *katA* in the *oxyR* mutant is most likely responsible for the lower biosensor oxidation under H_2_O_2_ stress [34], [50]. This hypothesis was supported by the very fast response of *katA* mutant cells to 40 mM H_2_O_2_ stress, resulting in fully oxidation of the biosensor due to the lack of H_2_O_2_ detoxification in the absence of KatA (Fig. 6**C**). Exposure of *katA* mutant cells to 40 mM H_2_O_2_ might cause enhanced oxidation of MSH to MSSM leading to full biosensor oxidation with no recovery of the reduced state. In contrast, kinetic biosensor measurements under H_2_O_2_ stress revealed only slightly increased oxidation in the *tpx* mutant while the *mpx* mutant showed the same oxidation increase like the wild type (Fig. 6**DE**). However, the H_2_O_2_ response of the *mpx tpx* mutant was similar compared to the wild type, indicating that Tpx and Mpx do not contribute significantly to H_2_O_2_ detoxification during exposure to high levels of 40 mM H_2_O_2_ stress, while KatA plays the major role (Fig. 6**F**). The small oxidation increase in the *tpx* mutant might indicate additional roles of Tpx for detoxification of low levels of H_2_O_2_ as found in previous studies [51]. Altogether, our studies on the kinetics of the Mrx1-roGFP2 biosensor response under H_2_O_2_ stress support that KatA plays the most important role in H_2_O_2_ detoxification in *C. glutamicum*.

**Fig. 7:**
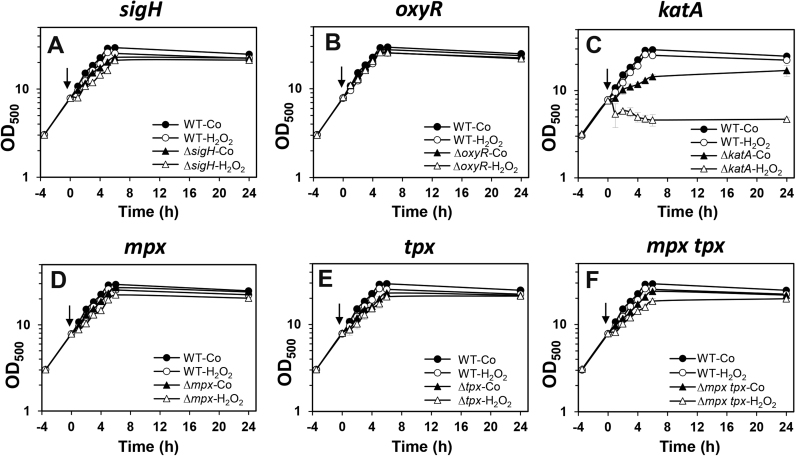
**H**_**2**_**O**_**2**_**sensitivity of*****C. glutamicum*****mutants deficient for redox-regulators (OxyR, SigH) or antioxidant enzymes (KatA, Mpx, Tpx).** The growth of various mutants with deletions of redox-sensitive regulators and antioxidant systems was compared after exposure to 80 mM H_2_O_2_, including ∆*sigH***(A),** ∆*oxyR***(B)***,* ∆*katA***(C)***,* ∆*mpx***(D)***,* ∆*tpx***(E)***,* ∆*mpx tpx* mutants **(F)**. Only the absence of KatA resulted in a strong H_2_O_2_ sensitive phenotype, while all other mutants were not affected by 80 mM H_2_O_2_ similar as the wild type. Mean values and SEM of three independent experiments are shown in all graphs. The time points of H_2_O_2_ exposure during the growth curves are set to ‘0’ and denoted with arrows. The control (Co) shows the growth curve of the *C. glutamicum* wild type and mutant strains without H_2_O_2_ stress exposure.

To correlate increased biosensor responses under H_2_O_2_ stress to peroxide sensitive phenotypes, we compared the growth of the wild type and mutants after exposure to 80 mM H_2_O_2_ (Fig. 7). Exposure of the wild type to 80 mM H_2_O_2_ did not significantly affect the growth rate indicating the high level of H_2_O_2_ resistance in *C. glutamicum*. Of all mutants, only the *katA* mutant was significantly impaired in growth under non-stress conditions and lysed after exposure to 80 mM H_2_O_2_ (Fig. 7**C**). In contrast, deletions of *sigH, oxyR, tpx* and *mpx* did not significantly affect the growth under control and H_2_O_2_ stress conditions (Fig. 7**AB, DE**). However, we observed a slightly decreased growth rate of the *mpx tpx* mutant in response to 80 mM H_2_O_2_ stress supporting the residual contribution of thiol-dependent peroxiredoxins in the peroxide stress response (Fig. 7**F**). Overall, the growth curves are in agreement with the biosensor measurements indicating the major role of KatA for detoxification of high levels of H_2_O_2_ and the recovery of cells from oxidative stress.

### 3.4 Single cell measurements of *E*_MSH_ changes under H_2_O_2_ stress using confocal imaging

**Fig. 8:**
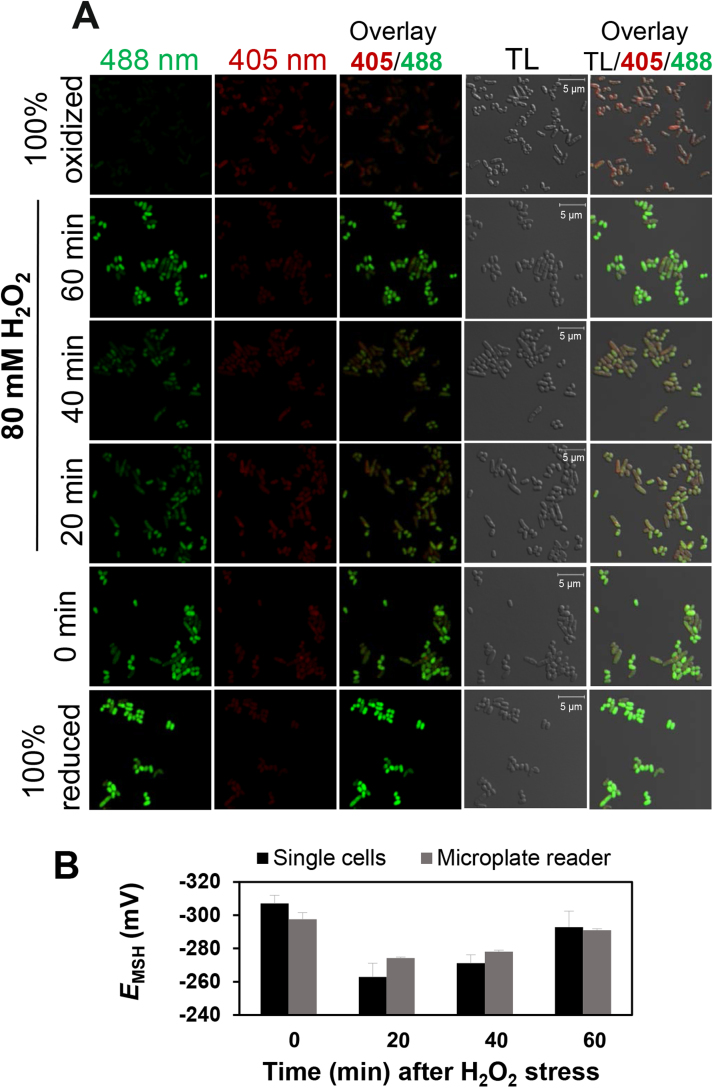
**Live-imaging of Mrx1-roGFP2 fluorescence changes in*****C. glutamicum*****wild type under H**_**2**_**O**_**2**_**stress at the single cell level. (A)***C. glutamicum* wild type cells expressing Mrx1-roGFP2 were challenged with 80 mM H_2_O_2_ for 20–60 min, blocked with 10 mM NEM and visualized by confocal laser scanning microscopy (CLSM). The time point ‘0’ indicates the untreated *C. glutamicum* wild type sample. Fully reduced and oxidized control samples were obtained after treatment of cells with 10 mM DTT and 10 mM diamide, respectively. Fluorescence intensities at the 405 and 488 nm excitation maxima are false-colored in red and green, respectively. Emission was measured between 491 and 580 nm. The oxidation degree is shown as overlay images of the transmitted light (TL)/405/488 channels. Images were analyzed by Zen software and Fiji/ ImageJ at separate channels. **(B)** The intracellular *E*_MSH_ was calculated based on the 405/488 nm excitation ratio of *C. glutamicum* Mrx1-roGFP2 cells after H_2_O_2_ treatment using confocal imaging and microplate reader measurements. Mean values and SEM of three independent experiments are shown. Bars, 5 µm.

To verify the biosensor response under H_2_O_2_ stress in *C. glutamicum* at the single cell level, we quantified the 405/488 nm fluorescence excitation ratio in *C. glutamicum* cells expressing stably integrated Mrx1-roGFP2 using confocal laser scanning microscopy (CLSM) **(**Fig. 8**A)**. For control, we used fully reduced and oxidized *C. glutamicum* cells treated with DTT and diamide, respectively. In the confocal microscope, most cells exhibited similar fluorescence intensities at the 405 and 488 nm excitation maxima, respectively, indicating that the Mrx1-roGFP2 biosensor was equally expressed in 99% of cells. Fully reduced and untreated *C. glutamicum* control cells exhibited a bright fluorescence intensity at the 488 nm excitation maximum which was false-colored in green, while the 405 nm excitation maximum was low and false-colored in red (Fig. 8**A**). In agreement with the microplate reader results, the basal *E*_MSH_ was highly reducing and calculated as −307 mV for the single cell population (Fig. 8**B,** Table S4). Treatment of cells with 80 mM H_2_O_2_ for 20 min resulted in a decreased fluorescence intensity at the 488 nm excitation maximum and a slightly increased signal at the 405 nm excitation maximum, causing an oxidative shift of *E*_MSH_. Specifically, the *E*_MSH_ of control cells was increased to −263 mV after 20 min H_2_O_2_ treatment. The recovery phase could be also monitored at the single cell level after 40 and 60 min of H_2_O_2_ stress, as revealed by the regeneration of reduced *E*_MSH_ of −271 mV and −293 mV, respectively (Fig. 8**B,** Table S4). The oxidative *E*_MSH_ shift after H_2_O_2_ treatment and the recovery of reduced *E*_MSH_ were comparable between the microplate reader measurements and confocal imaging (Fig. 8**B**). This confirms the reliability of biosensor measurements at both single cell level and for a greater cell population using the microplate reader.

## 4 Discussion

Here, we have successfully designed the first genome-integrated Mrx1-roGFP2 biosensor that was applied in the industrial platform bacterium *C. glutamicum* which is of high biotechnological importance. During aerobic respiration and under industrial production processes, *C. glutamicum* is frequently exposed to ROS, such as H_2_O_2_. Thus, *C. glutamicum* is equipped with several antioxidant systems, including MSH and the enzymatic ROS-scavengers KatA, Mpx and Tpx. Moreover, Mpx and Tpx are dependent on the MSH cofactor required for recycling during recovery from oxidative stress [16], [21], [22]. The kinetics of H_2_O_2_ detoxification has been studied for catalases and peroxiredoxins in many different bacteria. However, the roles of many H_2_O_2_ detoxification enzymes are unknown and many seem to be redundant and not essential [49]. There is also a knowledge gap to which extent the H_2_O_2_ detoxification enzymes contribute to the reduced redox balance under aerobic growth conditions and under oxidative stress.

Thus, we applied this stably integrated Mrx1-roGFP2 biosensor to measure dynamic *E*_MSH_ changes to study the impact of antioxidant systems (MSH, KatA, Mpx, Tpx) and their major regulators (OxyR, SigH) under basal conditions and ROS exposure. The basal *E*_MSH_ was highly reducing with ~-296 mV during the exponential growth and stationary phase in *C. glutamicum* wild type, but maintained reduced also in the *katA*, *mpx* and *tpx* mutants. In contrast, the probe was strongly oxidized in *mshC* and *mtr* mutants indicating the major role of MSH for the overall redox homeostasis under aerobic growth conditions. While the enzymatic ROS scavengers KatA, Mpx and Tpx did not contribute to the reduced basal level of *E*_MSH_ during the growth, the catalase KatA was essential for efficient H_2_O_2_ detoxification and the recovery of the reduced *E*_MSH_ under H_2_O_2_ stress. In contrast, both MSH-dependent peroxiredoxins Tpx and Mpx did not play a significant role in the H_2_O_2_ defense and recovery from stress, which was evident in the *tpx mpx* double mutant. These results were supported by growth phenotype analyses, revealing the strongest H_2_O_2_-sensitive growth phenotype for the *katA* mutant, while the growth of the *mpx tpx* double mutant was only slightly affected under H_2_O_2_ stress. These biosensor and phenotype results clearly support the major role of the catalase KatA for H_2_O_2_ detoxification.

Since expression of *katA* is controlled by the OxyR repressor, we observed even a lower H_2_O_2_ response of the *oxyR* mutant, due to the constitutive derepression of *katA* as determined previously [34]. In contrast, the *sigH* mutant showed an enhanced basal *E*_MSH_ during aerobic growth, since SigH controls enzymes for MSH biosynthesis and recycling (MshA, Mca, Mtr) which contribute to reduced *E*_MSH_ [29], [32]. However, the *sigH* mutant was not impaired in its H_2_O_2_ response of Mrx1-roGFP2, since H_2_O_2_ detoxification is the role of KatA. Thus, we have identified unique roles of SigH and Mtr to control the basal *E*_MSH_ level, while OxyR and KatA play the major role in the recovery of reduced *E*_MSH_ under oxidative stress.

In previous work, the kinetics for H_2_O_2_ detoxification by catalases and peroxiredoxins was been measured using the unfused roGFP2 biosensor in the Gram-negative bacterium *Salmonella* Typhimurium [52]. The deletion of catalases affected the detoxification efficiency of H_2_O_2_ strongly, while mutations in peroxidases (*ahpCF*, *tsaA*) had only a minor effect on the H_2_O_2_ detoxifying power. These results are consistent with our data and previous results in *E. coli*, which showed that catalases are the main H_2_O_2_ scavenging enzymes at higher H_2_O_2_ concentrations, while peroxidases are more efficient at lower H_2_O_2_ doses [53]. The reason for the lower efficiency of H_2_O_2_ detoxification by peroxidases might be due to low NAD(P)H levels under oxidative stress that are not sufficient for recycling of oxidized peroxidases under high H_2_O_2_ levels [53]. Overall, these data are in agreement with our Mrx1-roGFP2 measurements in the *katA, tpx* and *mpx* mutants in *C. glutamicum*.

However, *C. glutamicum* differs from *E. coli* by its strong level of H_2_O_2_ resistance since *C. glutamicum* is able to grow with 100 mM H_2_O_2_ and the biosensor did not respond to 10 mM H_2_O_2._ In contrast, 1–5 mM H_2_O_2_ resulted in a maximal roGFP2 biosensor response with different detoxification kinetics in *E. coli* [52]. Since the high H_2_O_2_ resistance and detoxification power was attributed to the catalases, it will be interesting to analyze the differences between activities and structures of the catalases of *C. glutamicum* and *E. coli*. Of note, due to its remarkable high catalase activity, KatA of *C. glutamicum* is even commercially applied at Merck (CAS Number 9001-05-2). However, the structural features of KatA that are responsible for its high catalase activity are unknown.

While our biosensor results confirmed the strong H_2_O_2_ detoxification power of the catalase KatA [51], the roles of the peroxiredoxins Mpx and Tpx for H_2_O_2_ detoxification are less clear in *C. glutamicum*. Both Tpx and Mpx were previously identified as *S*-mycothiolated proteins in the proteome of NaOCl-exposed *C. glutamicum* cells [16]. *S*-mycothiolation inhibited Tpx and Mpx activities during H_2_O_2_ detoxification *in vitro*, which could be restored by the Trx and Mrx1 pathways [16], [21], [22]. Moreover, Tpx displayed a gradual response to increasing H_2_O_2_ levels and was active as Trx-dependent peroxiredoxin to detoxify low doses H_2_O_2_ while high levels H_2_O_2_ resulted in overoxidation of Tpx [51]. Overoxidation of Tpx caused oligomerization to activate the chaperone function of Tpx. Since *mpx* and *katA* are both induced under H_2_O_2_ stress, they were suggested to compensate for the inactivation of Tpx for detoxification of high doses of H_2_O_2._ Previous analyses showed that the *katA* and *mpx* mutants are more sensitive to 100–150 mM H_2_O_2_ [21], [22]. In our analyses, the *mpx* mutant was not more sensitive to 80 mM H_2_O_2_ and displayed the same H_2_O_2_ response like the wild type, while the *katA* mutant showed a strong H_2_O_2_ sensitivity and responded strongly to H_2_O_2_ in the biosensor measurements. Thus, our biosensor and phenotype results clearly support the major role of KatA in detoxification of high doses H_2_O_2_ *in vivo*.

Finally, we confirmed using confocal imaging further that the genomically expressed Mrx1-roGFP2 biosensor shows equal fluorescence in the majority of cells indicating that the biosensor strain is suited for industrial application to quantify *E*_MSH_ changes in *C. glutamicum* at the single cell level or under production processes. Previous Mrx1-roGFP2 biosensor applications involved plasmid-based systems which can result in different fluorescence intensities within the cellular population due to different copy numbers. Moreover, plasmids can be lost under long term experiments when the selection pressure is decreased due to degradation or inactivation of the antibiotics.

We also compared the fluorescence intensities of the plasmid-based expression of Mrx1-roGFP2 using the IPTG-inducible pEKEx2 plasmid with the stably integrated Mrx1-roGFP2 strain in this work (Fig. S1). Using confocal imaging, the plasmid-based Mrx1-roGFP2 biosensor strain showed only roGFP2 fluorescence in < 20% of cells, while the genomically expressed biosensor was equally expressed and fluorescent in 99% of cells. The integration of the Mrx1-roGFP2 biosensor was performed into the *cg1121–1122* intergenic region and the biosensor was expressed from the strong P_*tuf*_ promoter using the pK18*mobsacB* construct designed previously for an Lrp-biosensor to measure L-valine production [54]. Previous live cell imaging using microfluidic chips revealed that only 1% of cells with the Lrp-biosensor were non-fluorescent due to cell lysis or dormancy [54]. Thus, expression of roGFP2 fusions from strong constitutive promoters should circumvent the problem of low roGFP2 fluorescence intensity after genomic integration. The advantage and utility of a stably integrated Grx1-roGFP2 biosensor has been also recently demonstrated in the malaria parasite *Plasmodium falciparum* which can circumvent low transfection frequency of plasmid-based roGFP2 fusions [55]. Moreover, quantifications using the microplate reader are more reliable, less time-consuming and reproducible with strains expressing genomic biosensors compared to measurements using confocal microscopy [55]. Thus, stably integrated redox biosensors should be the method of the choice for future applications of roGFP2 fusions to monitor redox changes in a greater cellular population.

In conclusion, in this study we designed a novel Mrx1-roGFP2 biosensor to monitor dynamic *E*_MSH_ changes in *C. glutamicum* during the growth, under oxidative stress and in mutants with defects in redox-signaling and H_2_O_2_ detoxification. This probe revealed the impact of Mtr and SigH to maintain highly reducing *E*_MSH_ throughout the growth and the main role of KatA and OxyR for efficient H_2_O_2_ detoxification and the regeneration of the redox balance. This probe is now available for application in engineered production strains to monitor the impact of industrial production of amino acids on the cellular redox state. In addition, the effect of genome-wide mutations on *E*_MSH_ changes can be followed in *C. glutamicum* in real-time during the growth, under oxidative stress and at the single cell level.
