# Supplementary material for: Stable integration of the Mrx1-roGFP2 biosensor to monitor dynamic changes of the mycothiol redox potential in Corynebacterium glutamicum
Source: Redox Biol. 2018 Nov 17;20:514–25. doi: 10.1016/j.redox.2018.11.012 (PMC6258114; doi:10.1016/j.redox.2018.11.012)
Supplement: Supplementary file 1 — Supplementary material. [file mmc1.pdf]

Table S1. Bacterial strains and plasmids

Bacterial strains	Description	Reference
Escherichia coli		
E. coli DH5α	F-φ80dlacZ Δ(*lacZYA-argF*) U169 deoRsupE44ΔlacU169 (f80lacZDM15) hsdR17 recA1 endA1 (rk- mk+) supE44gyrA96 thi-1 gyrA69 relA1	[1]
E. coli BL21(DE3) p*lysS*	F- ompT hsdS gal (rb- mb+) DE3(Sam7 Δnin5 lacUV5-T7 Gen1)	[1]
E. coli JM109	*end*A1, *rec*A1, *gyr*A96, *thi*, *hsd*R17 (rk–, mk+), *rel*A1, *sup*E44, *Δ(lac-proAB)*, F´ *tra*D36, *proAB*, *laqIq lacZ*ΔM15	[2]
Corynebacterium glutamicum		
C. glutamicum ATCC13032	wild type	
C. glutamicum Δ*mshC*	ATCC 13032 deletion of *mshC*	[3]
C. glutamicum Δ*mtr*	ATCC 13032 deletion of *mtr*	This study
C. glutamicum Δ*sigH*	ATCC 13032 deletion of *sigH*	[4]
C. glutamicum Δ*oxyR*	ATCC 13032 deletion of *oxyR*	[5]
C. glutamicum Δ*tpx*	ATCC 13032 deletion of *tpx*	[4]
C. glutamicum Δ*mpx*	ATCC 13032 deletion of *mpx*	[4]
C. glutamicum Δ*tpx mpx*	ATCC 13032 deletion of *tpx*, *mpx*	This study
C. glutamicum Δ*katA*	ATCC 13032 deletion of *katA*	This study
WT::P_{tuf}-*mrx1-roGFP2*	wild type with integrated P_{tuf}-*mrx1-roGFP2* into the intergenic region of *cg1121-cg1122*	This study
Δ*oxyR*::P_{tuf}-*mrx1-roGFP2*	Δ*oxyR* with integrated P_{tuf}-*mrx1-roGFP2* into the intergenic region of *cg1121-cg1122*	This study
Δ*mshC*::P_{tuf}-*mrx1-roGFP2*	Δ*mshC* with integrated P_{tuf}-*mrx1-roGFP2* into the intergenic region of *cg1121-cg1122*	This study
Δ*mtr*::P_{tuf}-*mrx1-roGFP2*	Δ*mtr* with integrated P_{tuf}-*mrx1-roGFP2* into the intergenic region of *cg1121-cg1122*	This study
Δ*sigH*::P_{tuf}-*mrx1-roGFP2*	Δ*sigH* with integrated P_{tuf}-*mrx1-roGFP2* into the intergenic region of *cg1121-cg1122*	This study
Δ*oxyR*::P_{tuf}-*mrx1-roGFP2*	Δ*oxyR* with integrated P_{tuf}-*mrx1-roGFP2* into the intergenic region of *cg1121-cg1122*	This study
Δ*tpx*::P_{tuf}-*mrx1-roGFP2*	Δ*tpx* with integrated P_{tuf}-*mrx1-roGFP2* into the intergenic region of *cg1121-cg1122*	This study
Δ*mpx*::P_{tuf}-*mrx1-roGFP2*	Δ*mpx* with integrated P_{tuf}-*mrx1-roGFP2* into the intergenic region of *cg1121-cg1122*	This study
Δ*tpx mpx*::P_{tuf}-*mrx1-roGFP2*	Δ*tpx mpx* with integrated P_{tuf}-*mrx1-roGFP2* into the intergenic region of *cg1121-cg1122*	This study
Δ*katA*::P_{tuf}-*mrx1-roGFP2*	Δ*katA* with integrated P_{tuf}-*mrx1-roGFP2* into the intergenic region of *cg1121-cg1122*	This study

WT:: pEKEx2-*mrx1-roGFP2*	wild type harboring inducible plasmid pEKEx2-*mrx1-roGFP2*	This study
**Plasmids**		
pET11b	*E. coli* expression plasmid	
PUCSP-P_{tuf}-*mrx1-roGFP2*	P_{tuf}-*mrx1-roGFP2* construct	This study
pK18*mobsacB*	*sacB, lacZα, mcs (km^R)*	[6]
pK18*mobsacB::Δtpx*	pK18*mobsacB::Δtpx*	[3]
pK18*mobsacB::ΔkatA*	pK18*mobsacB::ΔkatA*	This study
pK18*mobsacB::Δmtr*	pK18*mobsacB::Δmtr*	This study
pET11b-*mrx1-roGFP2*	pET11b-derivative for overexpression of His-tagged Mrx1-roGFP2	This study
pK18*mobsacB-int*	Km^R; plasmid for integration of foreign DNA into the intergenic region between *cg1121-cg1122* (*oriV$_{E.c}$*., *sacB*, *lacZ*α)	[7]
pK18*mobsacB-int*-P_{tuf}-*mrx1-roGFP2*	pK18*mobsacB* derivative for genomic integration of *P_{tuf}-mrx1-roGFP2* gene into the intergenic region of *cg1121-cg1122*	This study
pEKEx2	Kan^r; *E. coli–C. glutamicum* shuttle vector for regulated gene expression (P_{tac}*lacI*^{q} pBL1 *oriV$_{C.g.}$* pUC18 *oriV$_{E.c.}$)*	[8]
pEKEx2-*mrx1-roGFP2*	pEKEx2 derivative for IPTG-inducible *mrx1-roGFP2* gene expression	This study

Table S2. Oligonucleotide primers

Primer name	Sequence (5’ to 3’)
**Construction of pK18*mobsacB-int*-P*tuf-mrx1-roGFP2***	
roGFP2-FOR-NheI	CTAGCTAGCATGGTGAGCAAGGGCGAGGAG
roGFP2-REV-BamHI	CGCGGATCCTTAGTGATGGTGATGGTGATGCTTGTACAGCTCGTCCATGC
pk18_INT_Cg_Test_rev	AATCAATGAGCGCCGTGAAG
pk18_INT_Cg_Test_fwd	GGAAACTCAACGTCCACAAG
FUB_7_seq_wo_linker_fwd	CGTCGTTGACTGACTTAACC
FUB_8_seq_wo_linker_rev	AATCCTCGTGGAACTGGATG
**Construction of deletion mutants**	
JM33-katA_d1	AGGTCTCGAATTCCGTATTCGACGATGGATTTG (*Eco*RI)
JM34-katA_d2	AAATCAGAAGGACTTAAGCCCCTTTTATGAGGGTTACCTATG
JM35-katA_d3	GGCTTAAGTCCTTCTGATTT
JM36-katA_d4	AGGTCTCCGATCGCCGTGCCAGGAATTGCAAAG (*Pvu*I)
cg2194_mtr_d1	GGTGGTGAATTCCAGTCATGGCTGGCCAAGCA (*Eco*RI)
cg2194_mtr_d2	ACTTACGGCTGCGTAAGCTTCTGGCTGCTCAGACATAAGA
cg2194_mtr_d3	AAGCTTACGCAGCCGTAAGT
cg2194_mtr_d4	GGTGGTGGATCCGAGAAGCCGGATGAGAGGTT (*Bam*HI)
**Chromosomal deletion test primer**	
tpx_TEST_1	GAAGTTGTGGCGTTCTTCGT
tpx_TEST_2	ACTCACTAGGCGCACTGAAT
mpx_TEST_1	TGCAGCAGCTGTTCCTTCAA
mpx_TEST_2	AATCGCCGTAGGAGAATCCA
katA_TEST1	TAGCCCAACACTTGGGTGAG
katA_TEST2	CTGGAGTTGCACAGGCTTTTTG
mtr_TEST1	TTACGGCGAATGGGACTTTG
mtr_TEST2	CGTGCTCTTCACCAAAGATG
**Construction of pEKEx2- *mrx1-roGFP2***	
pEKEx2-Cgmrx1-BamHI-For	TAGGGATCCATGAGCAACGTAACCATTTACG (*Bam*HI)
pEKEx2-roGFP2-KpnI-Rev	CTCGGTACCTTACTTGTACAGCTCGTCCATGC (*Kpn*I)

Table S3A. Basal MSH redox potential of *C. glutamicum* wild type and mutant strains along the growth curve at different time points

Time (h)	E_{MSH} (mV)								
	**WT**	**Δ*mshC***	**Δ*mtr***	**Δ*sigH***	**Δ*oxyR***	**Δ*tpx***	**Δ*mpx***	**Δ*tpx mpx***	**Δ*katA***
**5**	-297.6±1.6	-232.3±13.2	-277.2±2.9	-285.7±2.9	-310.8±13.9	-307.6±2.9	-291.5±2.2	-288.3±7.2	-298.5±2.7
**6**	-297.8±7.2	-247.1±10.1	-280.0±2.4	-288.1±8.3	-305.2±8.4	-312.9±1.7	-291.1±2.5	-292.6±8.5	-299.2±5.0
**7**	-292.7±4.0	-238.0±14.1	-279.0±3.4	-286.9±6.1	-306.2±10.0	-313.5±4.9	-293.3±3.5	-304.6±11.6	-298.8±1.3
**8**	-298.7±7.4	-246.7±14.0	-278.5±2.2	-285.3±5.2	-299.8±13.4	-311.4±1.6	-292.4±6.5	-297.2±10.2	-304.5±1.5
**9**	-304.2±3.9	-251.5±22.7	-286.8±1.1	-287.5±3.3	-303.9±9.4	-308.2±2.8	-297.1±1.3	-307.5±4.4	-308.5±4.6
**10**	-294.5±6.4	-242.8±10.5	-278.8±1.8	-286.5±1.9	-307.2±6.8	-309.7±5.9	-302.8±13.1	-310.5±5.2	-300.5±1.7
**11**	-283.9±4.4	-257.8±5.2	-288.6±3.0	-284.4±3.6	-310.1±8.9	-303.7±1.7	-293.1±2.9	-309.2±10.3	-302.8±2.3
**12**	-296.1±1.5	-251.5±3.0	-287.1±3.7	-285.9±1.0	-302.9±3.9	-301.1±3.1	-301.9±1.2	-318.4±8.8	-302.6±11.3

Table S3B. Average value of the basal MSH redox potential of *C. glutamicum* wild type and mutant strains along the growth curve

C. glutamicum **Mrx1-roGFP2**	E_{MSH} **(mV)**
Wild type	-295.7 ± 2.2
Δ*mshC*	-246.0 ± 8.1
Δ*mtr*	-280.2 ± 4.6
Δ*sigH*	-286.3 ± 1.2
Δ*oxyR*	-305.8 ± 3.7
Δ*tpx*	-308.5 ± 4.1
Δ*mpx*	-295.4 ± 4.3
Δ*tpx mpx*	-302.1 ± 11.9
Δ*katA*	-302.0 ± 5.9

Table S4. Effect of 80 mM H_2O_2 on E_{MSH} changes as revealed by confocal imaging of single cells and microplate reader measurements

**Time (min) after 80 mM H_2O_2 stress**	E_{MSH} **(mV)**	
	**CLSM of single cells**	**Microplate reader**
0	-307.1 ± 4.9	-297.6 ± 3.9
20	-262.9 ± 8.2	-274.2 ± 0.6
40	-271.2 ± 5.1	-278.1 ± 0.9
60	-292.8 ± 9.6	-290.9 ± 1.0

Figure S1

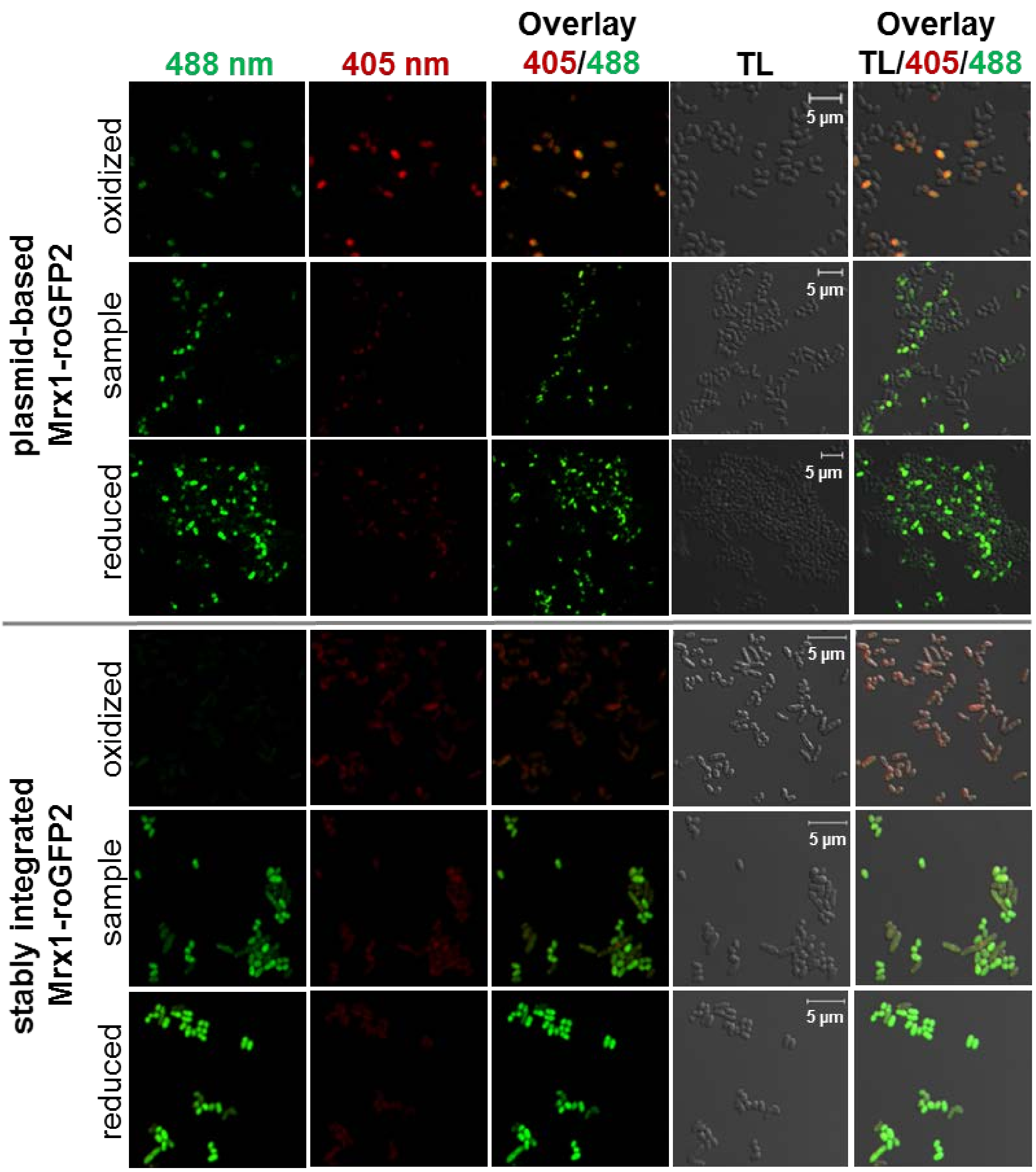

Figure S1: Live-imaging of *C. glutamicum* wild type with stably integrated and ectopically plasmid-encoded of Mrx1-roGFP2 biosensor. The *C. glutamicum* strains with stably integrated Mrx1-roGFP2 and ectopically plasmid-encoded Mrx1-roGFP2 were cultivated in HI medium and analyzed after 48 hours of growth. In addition, 1mM IPTG was added for 24 hours to *C. glutamicum* cells with plasmid-encoded Mrx1-roGFP2. Fluorescence intensities at the 405 and 488 nm excitation maxima are false-coloured in red and green, respectively. Emission was measured between 491 and 580 nm. The oxidation degree of Mrx1-roGFP2 expressing cells is shown as overlay images of the TL/405/488 channels. Images were analyzed by Zen software and Fiji/ ImageJ at separate channels. Bars, 5 µm.

SUPPLEMENTARY REFERENCES

[1] F.W. Studier, B.A. Moffatt, Use of bacteriophage T7 RNA polymerase to direct selective high-level expression of cloned genes, J Mol Biol 189(1) (1986) 113-30.

[2] C. Yanisch-Perron, J. Vieira, J. Messing, Improved M13 phage cloning vectors and host strains: nucleotide sequences of the M13mp18 and pUC19 vectors, Gene 33(1) (1985) 103-19.

[3] B.K. Chi, T. Busche, K. Van Laer, K. Bäsell, D. Becher, L. Clermont, G.M. Seibold, M. Persicke, J. Kalinowski, J. Messens, H. Antelmann, Protein S-mycothiolation functions as redox-switch and thiol protection mechanism in *Corynebacterium glutamicum* under hypochlorite stress, Antioxid Redox Signal 20(4) (2014) 589-605.

[4] B.K. Chi, T. Busche, K. Van Laer, K. Basell, D. Becher, L. Clermont, G.M. Seibold, M. Persicke, J. Kalinowski, J. Messens, H. Antelmann, Protein S-mycothiolation functions as redox-switch and thiol protection mechanism in *Corynebacterium glutamicum* under hypochlorite stress, Antioxidants & redox signaling 20(4) (2014) 589-605.

[5] J. Milse, K. Petri, C. Rückert, J. Kalinowski, Transcriptional response of *Corynebacterium glutamicum* ATCC 13032 to hydrogen peroxide stress and characterization of the OxyR regulon, J Biotechnol 190 (2014) 40-54.

[6] A. Schäfer, A. Tauch, W. Jäger, J. Kalinowski, G. Thierbach, A. Pühler, Small mobilizable multi-purpose cloning vectors derived from the *Escherichia coli plasmids* pK18 and pK19: selection of defined deletions in the chromosome of *Corynebacterium glutamicum*, Gene 145(1) (1994) 69-73.

[7] A.M. Nanda, A. Heyer, C. Kramer, A. Grünberger, D. Kohlheyer, J. Frunzke, Analysis of SOS-induced spontaneous prophage induction in *Corynebacterium glutamicum* at the single-cell level, J Bacteriol 196(1) (2014) 180-8.

[8] B.J. Eikmanns, E. Kleinertz, W. Liebl, H. Sahm, A family of *Corynebacterium glutamicum/Escherichia coli* shuttle vectors for cloning, controlled gene expression, and promoter probing, Gene 102(1) (1991) 93-98.
